# Integrative DNA methylation and transcriptome analysis reveal cell-type specific patterns in response to elevated allostatic load

**DOI:** 10.1080/15592294.2025.2578552

**Published:** 2025-10-29

**Authors:** O Emery, C Carmeli, S Gonseth-Nusslé, Rp Juster, C Kinnaer, D Nanchen, S Nusslé, S Stringhini, Jd Chamberlain

**Affiliations:** a Department of Health Promotion and Prevention (DPSP), Unisanté, University Center for Primary Care and Public Health, Lausanne, Switzerland; b Department of Epidemiology and Health Systems (DESS), Unisanté, University Center for Primary Care and Public Health, Lausanne, Switzerland; c Population Health Laboratory (#PopHealthLab), University of Fribourg, Fribourg, Switzerland; d Genknowme, Epalinges, Switzerland; e Department of Psychiatry and Addiction, University of Montreal, Montreal, Quebec, Canada; f School of Population and Public Health and Edwin S.H. Leong Centre for Healthy Aging, Faculty of Medicine, University of British Columbia, Vancouver, Canada; g Unit of Population Epidemiology, Division of Primary Care Medicine, Geneva University Hospitals & Faculty of Medicine, University of Geneva, Geneva, Switzerland

**Keywords:** Allostatic load, epigenetics, stress, methylation, SKIPOGH, transcriptomics

## Abstract

Allostatic load (AL) is a measure of the body’s multi-systemic physiological dysregulation in response to chronic stress and life events. High AL has been associated with poor long-term health outcomes such as cardiovascular disease and mortality. DNA methylation (DNAm) is an epigenetic mechanism involving both genes and environmental factors and contributes to gene expression regulation. Hence, changes in AL can possibly be reflected in DNAm and gene expression differences and leveraging epigenetic and transcriptomic data together can help elucidate the underlying biological processes involved. To assess differential DNAm and gene expression between high and low AL in a cell-type specific manner, bulk DNAm and transcriptome signals from whole blood samples of 429 individuals from the Swiss Kidney Project On Genes in Hypertension (SKIPOGH) cohort were first deconvoluted into cell-type specific signals for six blood cell types using tensor composition analysis (TCA) and the software CIBERSORTx. For each cell type, DNAm associated with gene expression changes was then determined in high (*N* = 126) vs low (*N* = 303) AL groups. A total of 263 CpG-gene pairs were identified across all cell types, corresponding to 250 unique CpGs and 138 unique differentially methylated genes (DMGs). Several immune processes were enriched among downregulated genes of CD8 T and B cells, suggesting an impairment of the immune response, which is compatible with high AL. These findings highlight the importance of using cell-specific signals in DNAm and transcriptome analyses and may contribute to identify AL biomarkers and/or potential therapeutic targets.

## GRAPHICAL ABSTRACT

**Figure:**
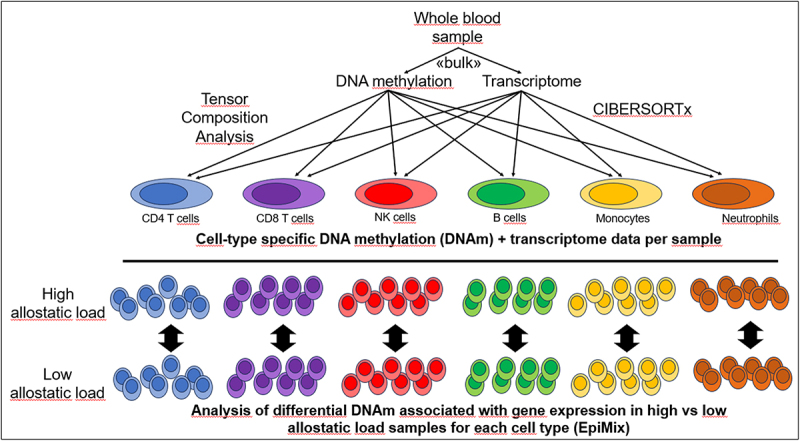

## Introduction

The ability to achieve stability through change, or allostasis, is critical for survival [1]. The core of the brain’s and the body’s response to a challenge consists of two parts: first to turn on an allostatic response that starts a complex adaptative cascade and, second, to turn off this response once the challenge is over [2]. Allostatic load (AL) refers to the cumulative burden due to chronic stress and life events on our brains and bodies, which could predispose the organism to disease. If environmental challenges surpass an individual’s ability to cope, allostatic overload follows [3,4]. The AL score is a composite measure integrating several biomarkers classified ranging across the cardiovascular, immune, neuroendocrine and metabolic systems [5]. High allostatic load has been previously associated with an increased mortality risk of 22% for all-cause mortality and 31% for cardiovascular disease mortality [6] as well as with poor physical and mental health outcomes [6,7].

The degree of adaptability of neurochemical stress response systems and the function of the neural circuitry involved in stress responses are determined by complex interactions between the genetic make-up of individuals and their personal history of exposure to environmental stressors [8]. In this context, epigenetics, i.e., the study of changes in gene expression that take place without modification of the DNA sequence, shows promise in further elucidating how stress responses are mediated. In fact, epigenetic modifications integrate both genetic and environmental components, and as such different epigenetic states may reflect how well individuals cope with stress [9]. DNA methylation is one of the most studied epigenetic modifications in humans that generally occurs at the cytosine-guanine dinucleotides sequences named ‘CpG sites.’ It involves the addition of a methyl group to DNA and plays an important role in regulating gene expression and many biological functions. Previous analyses of DNA methylation patterns have demonstrated the ability to distinguish healthy from diseased patients [10–12], and to quantify the exposure to environmental stressors such as alcohol or tobacco smoke [13,14]. Specific CpG sites may play a role in the adaptability of the stress response by regulating the expression of the genes related to those sites or act as a biomarker of allostatic load. Consistent with this, one recent study described the creation and validation of an epigenetic signature of AL, which correlated well with phenotype-based AL scores [15]. In addition, studies have shown that specific DNA methylation patterns are associated to psychological stress [16–18] and post-traumatic stress disorder [19–22]. Other studies regarding DNA methylation in the context of AL have considered accelerated aging using epigenetic clocks – estimators of biological age based on the methylation levels of a specific set of CpGs – but without analyzing the majority of CpG methylation values, even when data are available [23–25]. Beyond DNA methylation, other epigenetic mechanisms have been shown to be altered in response to diverging stress responses in murine models. These include post-translational histone modifications such as: decreased histone acetylation at the promoter of the glucocorticoid receptor in the hippocampus of adult offspring rats exposed to low maternal care [26], modified chromatin accessibility in the ventral hippocampus of a mouse model of acute stress in response to double-hit acute stress [27], and altered microRNA in the sperm of mice in response to traumatic stress in early life affecting behavioral and metabolic responses of their progeny [28].

While there is accumulating evidence that the methylation status of specific CpG sites is associated with environmental exposures, as in epigenome-wide association studies (EWAS), the link to gene expression is often implicit and not formally tested [29]. To do so, the simultaneous measurement of all transcripts in the genome, or transcriptome, must be obtained and analyzed in relation to CpG methylation levels. In addition, most studies do not consider the variation in methylation levels across the different cell types but only variation in cell composition. This limitation can be addressed using tensor composition analysis (TCA), an in-silico cell sorting, computational method that can extract cell specific methylomes from bulk tissue data [30]. The objective of this study was therefore to leverage signal deconvolution techniques to identify genes and pathways involved in allostatic load modulation across six different cell types using sample-matched methylation and transcriptome whole blood data.

## Material and methods

### Study population and design

This study used a subset of data from the Swiss Kidney Project on Genes in Hypertension (SKIPOGH). SKIPOGH is a multi-center, family- and population-based cohort study including Swiss adults (aged 18 years or older at study entry) of European ancestry with at least one first-degree family member also willing to participate [31,32]. Participants were recruited between 2009 and 2013 with a follow-up between 2012 and 2016 and data collected on sociodemographic information, health status (including medical history), physical activity, lifestyle and sleeping habits, and clinical and anthropometric exam results with DNA methylation data collected only for a subsample of participants from the follow-up [31,32]. Hence, the present study is restricted to information collected during follow-up.

### Data and sample selection

Of the original 1062 SKIPOGH participants, epigenetic (Illumina Inifinium EPIC Illumina Infinium MethylationEPIC v1.0 BeadChip array) and transcriptomic data [33] were available for 433 individuals. Laboratory procedures for RNA and DNA extraction and bisulfite conversion from whole blood samples, as well as the bioinformatic steps leading to the transcriptome measurements were described previously [33].

### DNA methylation data preparation

Raw methylation measurements were imported into R using the ‘minfi’ Bioconductor package with the read.metharray.exp function, preprocessed with the preprocessRaw function and background correction was performed using the bgcorrect.illumina function [34]. Quantile normalization was conducted using a discrete strategy using sub-distributions separated per probe type, methylation status and color channels using 12 duplicate reference samples. The getSex function of the R package minfi version 1.48.0 was used to confirm the sex attribution of all samples. Since four samples had the opposite sex based on their methylation data than what was reported, they were excluded from further analyses, resulting in a final number of 429 individuals for analyses.

### Cell type proportions based on transcriptomic data and transcriptome deconvolution

The online version of CIBERSORTx (https://cibersortx.stanford.edu/) was used to first estimate cell type proportions for each sample using the LM6 signature matrix which allows to distinguish six peripheral blood immune subsets: CD8 T cells, CD4 T cells, B cells, NK cells, Neutrophils and Monocytes [35]. CIBERSORTx is an analytical tool to impute gene expression profiles and provide an estimation of the abundances of member cell types in a mixed cell population, using gene expression data [36]. CIBERSORTx high-resolution mode was then run locally using a docker container to obtain one transcriptome per cell type for each sample, using the previously obtained cell type fractions and whole blood transcriptomes from each sample as inputs.

### Allostatic load, group assignments, and other variables

Allostatic load scores were retrieved from Petrovic et al. [37] for all samples. Briefly, 14 biological markers within six homeostatic dimensions were assessed: mean systolic blood pressure, mean diastolic blood pressure and heart rate (cardiovascular system); blood glucose, blood insulin, body mass index and waist-to-hip ratio (metabolism), 24 h urine cortisol (HPA); high-density lipoprotein cholesterol, total cholesterol and triglycerides (lipidic acids); serum uric acid and gamma glutamyltransferase (GGT, oxidative stress), and C-reactive protein (CRP, inflammation). Each biological marker was dichotomized (0/1) according to clinical thresholds identified in the literature, stratified by birth-assigned sex where applicable. The AL score was computed by summing the dichotomized values, resulting in a possible range from 0 to 8 for an individual. For the complete list of clinical thresholds used and corresponding references, please refer to supplementary material of Petrovic *et al*. [37].

Samples were attributed to the low AL group if their AL score was equal or less than two (corresponding to the median AL score value in our dataset and in the full dataset [37]) and to the high AL group if greater than two. The resulting AL scores are integers with many repeated values in the low range, leading to skewed group sizes despite using the median value as a cutoff. Therefore, given the potential influence of AL operationalization on results, in a sensitivity analysis, an alternative, continuous estimation of the AL score was used based on the z-score method. This estimation included the 14 biomarkers used previously, as well as 11 additional variables: mean dehydroepiandrosterone over 24 h (µg, corrected for creatine), mean androsterone over 24 h (µg, corrected for day vs night urine volume), IFNγ (pg/mL), TNFα (pg/mL), IL6 (pg/mL), IL1b (pg/mL), IL10 (pg/mL), lipoprotein a (mg/mL), ALAT (U/L), LDL cholesterol (mmol/L) and albumin (g/L). Z-score values for the different biomarkers were determined using the total SKIPOGH population. Absolute z-scores of the 25 biomarkers were then summed together to compute the allostatic load score. The cutoff value used to distinguish low (*N* = 322) from high (*N* = 107) AL groups was defined as the third quartile of summed scores in the subset under study (cutoff value = 0.8695).

Additional variables including age, sex, current smoker status (‘yes’ or ‘no’), educational level, and self-reported perceived stress were retrieved from questionnaires. Educational level in the current study corresponds to having finished secondary school or not and self-reported stress corresponds to the question: ‘On a scale from 1 to 10, what is your level of daily stress?’

### Tensor composition analysis (TCA)

The R package TCA version 1.2.1 [30] was used using cell type proportions provided by CIBERSORTx to deconvolve methylomes into one methylome per cell type per sample. In order to avoid spurious methylation differences solely due to sex chromosomes or not reliable probes, methylation probes from sex chromosomes (*N* = 19’627) as well as 53’057 cross reactive and polymorphic probes previously reported in [38] were removed from the methylation data before analysis.

In addition to cell type estimates, we used the reference-free algorithm ReFACTor [39] in order to account for further cell type composition variation, as ReFACTor was shown to improve correction over a reference-based approach in multiple whole-blood datasets [40]. We computed ReFACTor components taking as technical covariates the recruitment center and the first principal components of control probes for each sample. We used the tca function, thereby setting the statistical hypothesis that AL affects methylation levels. We used sex, age, smoking status, and first-degree family group number as biological covariates (c1 argument), and recruitment center and the first 10 principal components of control probes as technical covariates (c2 argument). Finally, cell type specific methylomes for each cell type were generated using the above TCA model with the ‘tensor’ function.

### Differential methylation associated with gene expression

The regular mode of the Bioconductor package EpiMix v1.4.0 [41] was used to identify differentially methylated CpGs significantly associated to gene expression separately for each of the six cell types between high and low AL groups. EpiMix takes as input sample matched transcriptomes and methylomes, and a list indicating to which group each sample belongs to, in our case high or low AL. This package determines if there are significant correlations between gene expression and methylation levels for at least a subset of the test samples (high AL) relative to normal samples (low AL samples). We first performed analyses for each cell type with the ‘correlation’ option set to ‘negative.’ This option tests whether the methylation level at each CpG site in at least a subset of samples in the high AL group is significantly negatively associated with the expression of the corresponding gene, i.e., hypermethylation corresponding to low gene expression and, conversely, hypomethylation corresponding to high gene expression. CpG sites for which subsets of both significantly hypo- and hypermethylated states are found among the test group (i.e., the high AL group) are termed ‘dual methylated.’ The significant results returned by EpiMix are termed ‘CpG-gene functional pairs.’

Negative correlations between methylation and gene expression levels are typically found in gene promoters whereas positive correlations are usually found in gene bodies [42]. Hence, since the Illumina EPIC arrays contains CpGs distributed along the entire genome including gene promoter, gene body and intergenic regions [38], we ran EpiMix a second time with the correlation option set to ‘positive’ in order to also determine functional CpG-gene pairs with positive correlations. Both negative and positive correlation results were combined to determine the full list of differentially methylated genes (DMGs) associated with gene expression. In order to be compatible with EpiMix, 52’699 of the 57’820 Ensembl gene IDs which include protein-coding and non-coding genes contained in the transcriptome data were converted to gene names using the R packages AnnotationDbi v1.64.1 [43] and EnsDb.Hsapiens.v86 v2.99.0 [44]. The remaining 5’121 Ensembl IDs which did not map to a gene name were excluded from the analysis. In addition, for each cell type specific transcriptomes, genes which did not contain expression values for at least one sample were removed prior to analysis with EpiMix.

### Gene expression direction in high vs low AL groups

To determine which genes were up- or downregulated in at least a subset of high AL samples for the different cell types, EpiMix gene-CpG functional pairs results from positive and negative correlations were first concatenated for each cell type, including a field indicating whether they originated from negative or positive correlations. Cell-specific results were filtered per gene name and, for genes associated with a single CpG, if the CpG was hypomethylated in negative correlation results or hypermethylated in positive correlation results, the gene was marked as upregulated. Conversely, if a gene was associated with a single CpG which was hypermethylated in negative correlation results or hypomethylated in positive correlation results, it was marked as downregulated. For genes associated with a single dual-methylated CpG (i.e., a CpG for which there was a subset of high AL samples for which it was hypomethylated and another hypermethylated), we selected the highest prevalence between hypo and hypermethylated samples and applied the previously described scheme to determine if the gene was up- or downregulated according to the type of correlation it came from. For genes associated with several CpGs, the same scheme was applied per CpG, and genes were considered as upregulated if all CpGs associated to a gene were upregulated and, conversely, downregulated if all CpGs associated to a gene were downregulated. For 8 genes out of 138, there was an inconsistency in the gene regulation direction (i.e., at least one CpG suggested gene regulation in the opposite direction than all other CpGs associated to the gene) and these genes were not included in further analyses.

### GO terms and KEGG pathways annotation and enrichment analyses

Enrichment analyses of GO terms for the biological processes, molecular functions, or cellular compartments ontologies and for KEGG pathways were performed with the Bioconductor package clusterProfiler version 4.10.1 [45] separately for each cell type further divided into upregulated and downregulated genes. For each gene subset, GO terms or KEGG pathways with adjusted p-value < 0.05 were considered as significantly enriched.

All data handling, analyses, and visualization were conducted in R statistical software version 4.3.0 [46] with Bioconductor version 3.18 [47].

## Results

### Study sub-population characteristics

Study characteristics stratified by high versus low AL status are provided in Table 1. Out of 429 selected individuals, 303 (70.6%) were assigned to the low AL group (mean AL score = 0.964, SD=± 0.79), and 126 (29.4%) to the high AL group (mean AL score = 3.833, SD=± 1.14). Differences between AL group status were observed for age, sex and all 14 biomarkers used to compute AL scores (p-value < 0.001), with a higher proportion of males and older average age in the high AL group compared to the low AL group (Table 1). Interestingly, perceived stress status was higher in the low allostatic load group, although this was marginally significant (p-value = 0.05). No differences were observed between AL groups, recruitment centers, smoking status, or proportion of individuals having completed high school (p-values > 0.05).

**Table 1.** Characteristics of individuals of the current study stratified by al group. Variables with an asterisk (*) indicate the 14 biomarkers used to compute the al score (see methods for details). Perceived stress corresponds to the question ‘on a scale from 1 to 10, what is your level of daily stress?’ Significant p-values (<0.05) from statistical tests are highlighted in bold.

Variable	Low allostatic load group (N = 303)	High allostatic load group (N = 126)	p-value	Test
AL score, mean (± SD)	0.964 (±0.790)	3.833 (±1.14)	**2.20e-16**	Mann-Whitney-Wilcoxon Test
Number of males	133 (43.9%)	79 (62.7%)	**5.45e-9**	Chi^2^
Age, mean (± SD)	50.27 (±17.84)	61.30 (±14.91)	**3.20e-9**	Mann-Whitney-Wilcoxon Test
Current smokers	71 (23.4%)	26 (20.6%)	0.61	Chi^2^
Body mass index* (± SD)	24.3 (±4.26)	29.34 (±5.08)	**2.20e-16**	Mann-Whitney-Wilcoxon Test
Systolic blood pressure* (± SD)	115 (±14.73)	130 (±17.24)	**7.31e-16**	Mann-Whitney-Wilcoxon Test
Diastolic blood pressure* (± SD)	72.26 (±8.06)	79.24 (±10.03)	**4.39e-11**	Mann-Whitney-Wilcoxon Test
Heart rate BPM* (± SD)	64.48 (±9.59)	69.56 (±12.13)	**0.00011**	Mann-Whitney-Wilcoxon Test
Insulin* mU/L (± SD)	5.89 (±3.64)	14.27 (±23.66)	**2.2e-16**	Mann-Whitney-Wilcoxon Test
Glucose* mmol/L (± SD)	4.89 (±0.56)	6.00 (±2.47)	**2.47e-13**	Mann-Whitney-Wilcoxon Test
Waist to hip ratio* (± SD)	0.88 (±0.07)	0.96 (±0.072)	**2.2e-16**	Mann-Whitney-Wilcoxon Test
Cortisol* µg/24 h (± SD)	115.12 (±67.51)	149.21 (±82.23)	**8.356e-6**	Mann-Whitney-Wilcoxon Test
Triglycerides* mmol/L (± SD)	0.85 (±0.43)	1.51 (±0.79)	**2.2e-16**	Mann-Whitney-Wilcoxon Test
HDL cholesterol* mmol/L (± SD)	1.58 (±0.43)	1.32 (±0.38)	**1.055e-8**	Mann-Whitney-Wilcoxon Test
Total cholesterol* mmol/L (± SD)	4.87 (±0.97)	5.27 (±1.19)	**0.001459**	Mann-Whitney-Wilcoxon Test
CRP* mg/L (± SD)	1.67 (±2.92)	4.15 (±8.08)	**1.476e-10**	Mann-Whitney-Wilcoxon Test
Uric acid* µmol/L (± SD)	299.8 (±72.65)	367.18 (±96.53)	**4.297e-12**	Mann-Whitney-Wilcoxon Test
GGT U/L* (± SD)	22.77 (±17.36)	47.15 (±42.04)	**4.144e-15**	Mann-Whitney-Wilcoxon Test
Perceived stress, mean (± SD)	4.46 (±2.16)	4.03 (±2.58)	**0.0491**	Mann-Whitney-Wilcoxon Test
Recruitment center				
Lausanne	118 (38.9%)	49 (38.9%)	0.8	Chi^2^
Geneva	142 (46.9%)	62 (49.2%)		
Bern	43 (14.2%)	15 (11.9%)		
Education				
Secondary school completed	135 (44.5%)	49 (38.8%)	0.33	Chi^2^

Cell type proportions were estimated for each sample based on the transcriptomes of the 429 samples under study (Supplementary Figure S1) and used to separate transcriptomes and methylomes per cell types (see Methods). Low and high AL samples showed statistically significant differences in proportions for CD4 T cells, B cells and Monocytes (p-value < 0.05, Supplementary Figure S2).

### Cell type specificity of differentially methylated genes associated to gene expression

A total of 263 functional CpG-gene pairs, corresponding to 250 unique CpGs and 138 unique genes (hereafter termed DMGs for ‘differentially methylated genes’) were identified in response to high AL when considering both negative and positive correlations between methylation levels and gene expression across all cell types (Supplementary Table S1). Of these, 173 functional CpG-gene corresponding to 163 unique CpGs and to 102 unique genes across all cell types were identified in EpiMix analyses set to detect negative correlations. An additional 90 functional CpG-gene pairs, corresponding to 87 unique CpGs and 56 unique genes were identified when assessing positive correlations. Twenty genes were present in both positive and negative correlation analyses of the same cell type and showed consistent gene expression direction.

**Figure 1.**
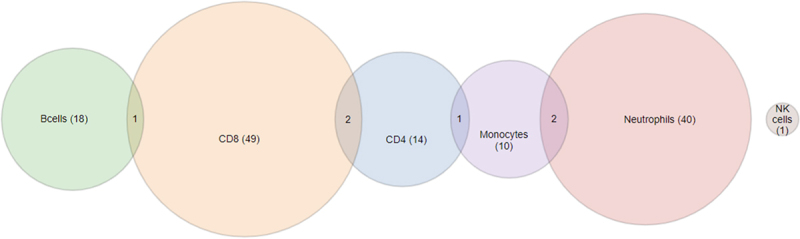
Number of differentially expressed genes associated to dna methylation differences between high and low allostatic groups in each of the six cell types studied show little overlap between cell types.

Considering DMGs separately per each cell type, CD8 T cells had the highest number of DMGs with a total of 52, neutrophils were second with 42 DMGs, B cells had 19 DMGs, 14 DMGs were identified for CD4 T cells, and 13 DMGs for monocytes (Figure 1). Certain DMGs were shared between the different cell types. For example, the RHOH gene was differentially methylated in both CD8 T and B cells, while NRCAM and NT5E (formerly known as CD73) were shared between CD4 and CD8 T cells. CES1 and CLEC4C were shared between monocytes and neutrophils. CD4 T and monocyte cells shared a single DMG, namely TSPYL5. NK cells contained a single DMG corresponding to PDGFRB. The majority of significant DMGs were specific to a given cell type with a small number of genes (6/138 corresponding to 4.3%) shared at most between two cell types, with 68% of DMGs found in either CD8 T cells (*N* = 52) or Neutrophils (*N* = 42).

Venn diagram showing the number of differentially expressed genes associated to DNA methylation differences between high and low allostatic groups in each of the six cell types studied. This includes both negative and positive correlations between methylation and gene expression levels. Numbers in parentheses indicate the number of genes which are differentially expressed specifically for the corresponding cell type indicated above. Numbers at the intersection of two circles indicate the number of differentially expressed genes which are shared between the cell types designated by the corresponding two circles.

### Gene regulation and significant CpGs methylation statuses vary between cell types

**Figure 2.**
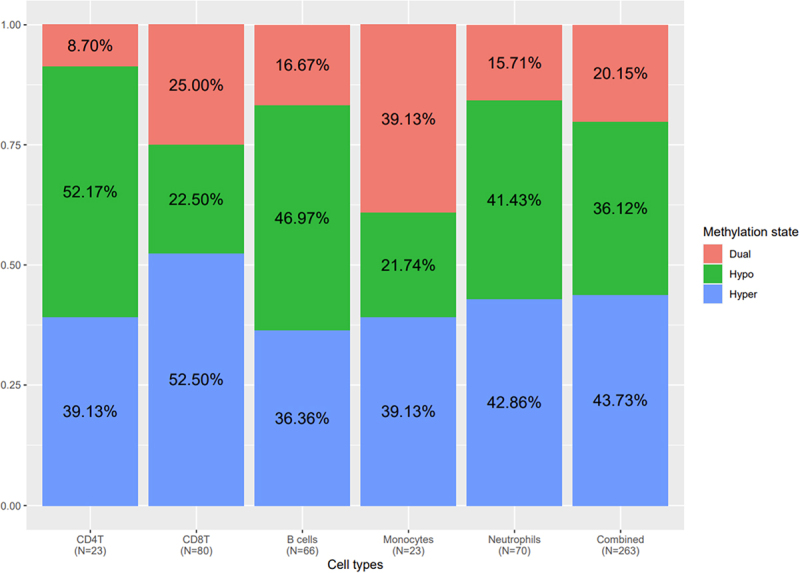
Proportion of the different methylation statuses of all CpG-gene functional pairs per cell type and combined.

The proportion of methylation status (i.e., hypo-, hyper- or dual-methylated) of statistically significant functional gene-CpG pairs by cell type are provided in Figure 2. When considering all significant CpGs from all cell types, most CpGs were hypermethylated (43.7%). In contrast to CD4 T and B cells, which had more hypomethylated CpGs, there were more hypermethylated CpGs in CD8 T cells (52.5% hypermethylated) and monocytes (39.1% hypermethylated). Neutrophils had similar proportions of hyper and hypomethylated CpGs (41.4% and 42.8%, respectively). Only one CpG was identified for NK cells, which was hypermethylated. Excluding NK cells, the proportion of dual methylated CpGs ranged from 8.7% for CD4 T cells to 39.1% for monocytes. Globally, different proportions for the three methylation statuses according to cell types were observed, which indicates cell type-specific global methylation patterns in response to high AL.

The proportion of CpG-gene functional pairs which were hypomethylated (green), hypermethylated (blue) or dual methylated (red) are shown for each cell type and for all cell types combined in the last column. The total number of CpG-gene functional pairs per cell type is indicated in the x-axis below cell types. Note that there can be multiple CpG-gene functional pairs per gene. NK cells had a single gene-CpG functional pair which was hypermethylated, and the corresponding column was not included in this figure.

**Figure 3.**
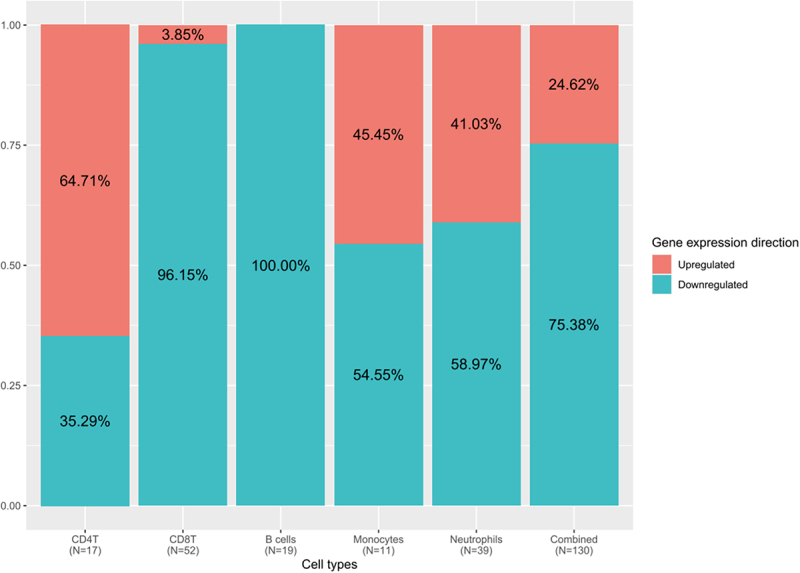
Gene expression direction for differentially methylated genes vary per cell type.

After predicting for each DMG from each cell type the direction of gene expression in high AL samples (see methods), bar plots were produced to display the proportion of up- and downregulated genes in the different cell types and when combining all of them (Figure 3). In the combined dataset consisting of 130 DMGs, 75.4% are downregulated and 24.6% are upregulated. As previously for methylation status patterns, differences were observed in the proportions of up- and downregulated genes in the different cell types. B cells and CD8 T cells had the highest proportions of downregulated genes (100% and 96.1%, respectively), followed by neutrophils (59%) and monocytes (54.5%). In contrast, CD4 T cells exhibited a higher proportion of upregulated genes (64.7%) than downregulated genes (35.3%). For NK cells, the only DMG was upregulated. Hence different cell types show varying patterns in gene expression.

The proportion of DMGs which were upregulated (red) or downregulated (cyan) are shown for each cell type and for all cell types combined in the last column. The total number of DMGs per cell type is indicated in the x-axis below cell types. NK cells had a single gene which was upregulated, and the corresponding column was not included on this figure.

### GO enrichment analyses by cell type and gene expression direction

For GO biological processes (BP), enriched terms were found for downregulated genes of CD8 T cells, downregulated genes of B cells and for upregulated genes in neutrophils (Figure 4a and Supplementary Table S2). Downregulated genes of CD8 T cells were enriched for BP terms linked to the adaptive immune response including T cell differentiation and activation, lymphocyte apoptotic process, signal transduction but also terms associated with vasculature development and angiogenesis as well as the forebrain generation of neurons. Downregulated genes of B cells were enriched for GO BP terms which were all linked to humoral immunity and shared the term ‘antigen receptor-mediated signaling pathway’ with enriched BP terms from downregulated genes of CD8 T cells. Upregulated genes from neutrophils yielded only one enriched GO BP term which was ‘cellular metabolic compound salvage.’

**Figure 4a.**
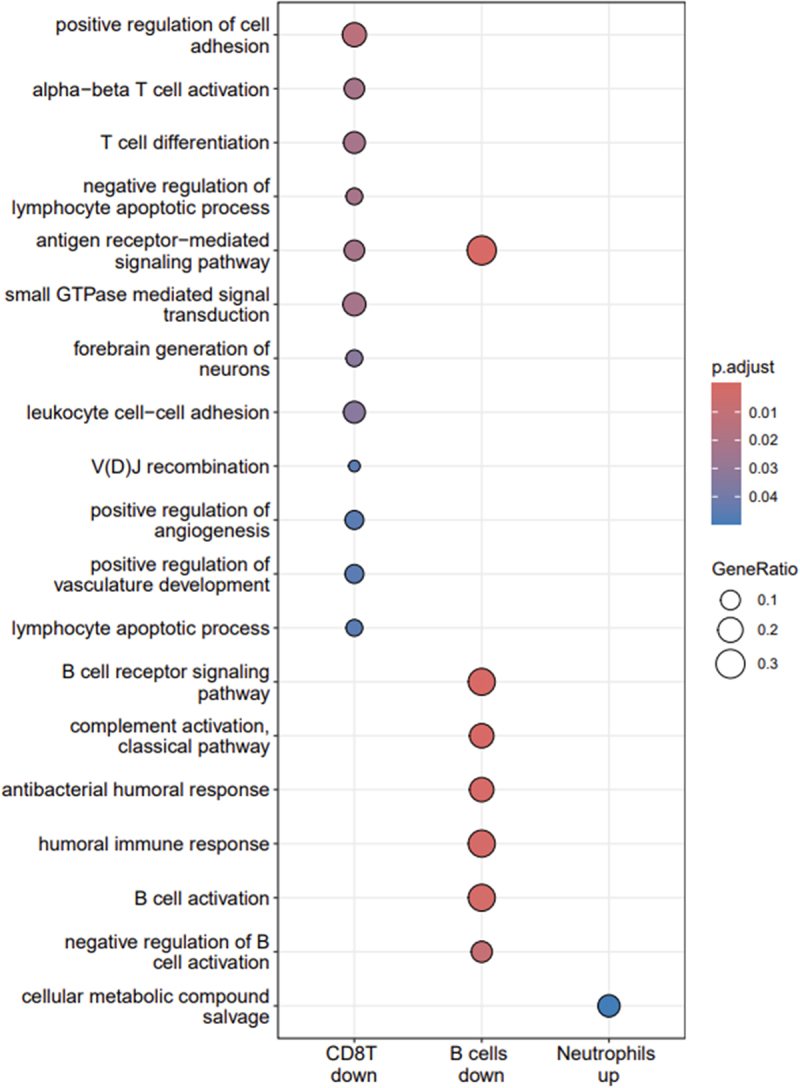
Enrichment of biological process go terms by cell type and down- or up-regulated genes.

Gene sets for which Gene Ontology (GO) biological processes were enriched are shown on the X-axis (including the corresponding cell type name followed by ‘down’ for downregulated and ‘up’ for upregulated genes) with the corresponding GO terms shown on the Y-axis. The sizes of the dots are proportional with the gene ratio which corresponds to the number of genes associated with a given term divided by the number of all genes associated with this term. The colors of dots indicate the level of significance of the adjusted p-value with a gradient from blue (marginally significant) to red (highly significant).

According to GO molecular function (MF) enrichments, over-represented terms downregulated genes in CD8 T cells include ‘ankyrin binding’ and ‘extracellular matrix binding,’ which are important for tissue integrity and cell communication (Figure 4b and Supplementary Table S2). For downregulated genes in B cells, two central molecular functions of the immune system in the recognition and defense against pathogens were enriched: ‘antigen binding’ and ‘immunoglobulin receptor binding.’ Downregulated genes in B cells were enriched for only ‘deacetylase activity.’ Upregulated genes in CD4 T cells were enriched for seven MF terms which play multiple roles in cell homeostasis, cell growth and response to external stimuli. Downregulated genes of B cells were the only gene subset enriched for terms of the ‘Cellular compartment’ GO ontology with two terms related to the immunoglobulin complex (‘Immunoglobulin complex, circulating’ and ‘IgG immunoglobulin complex’), ‘blood microparticle’ and ‘Wnt signalosome’ (Figure 4c and Supplementary Table S2). KEGG enrichment analyses revealed 13 pathways enriched in upregulated genes of CD4 T cells including several metabolic pathways (glutathione, glycerolipid, glycerophospholipid), pathways involved in drug metabolism and resistance, cancer-related pathways as well as a pathway linked to atherosclerosis and a phospholipase D signaling pathway (Figure 4d and Supplementary Table S2).

**Figure 4b.**
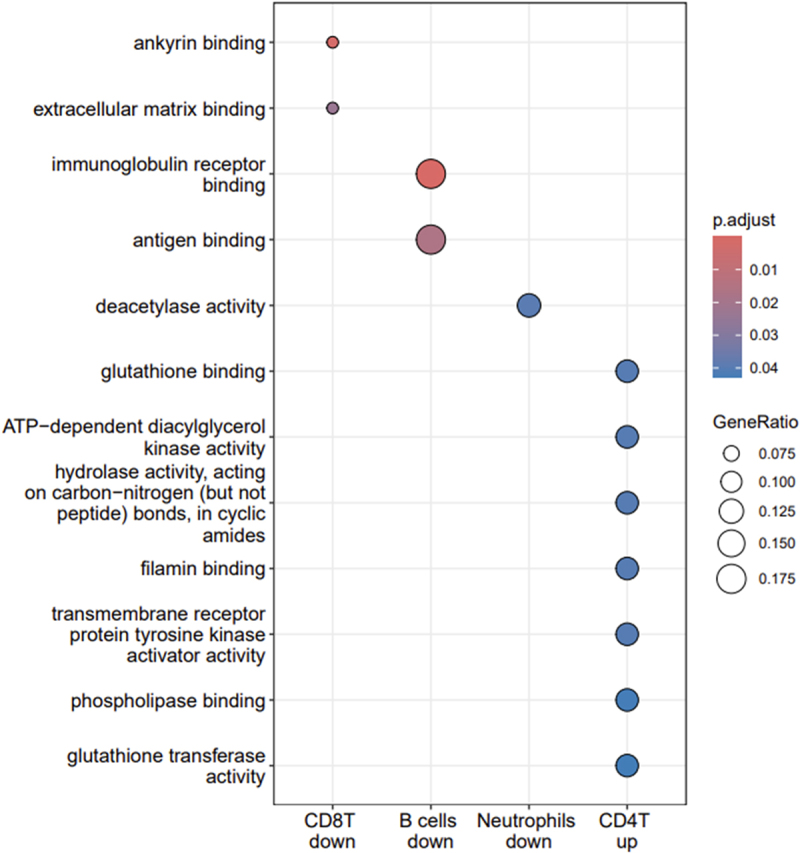
Enrichment of molecular function go terms by cell type and down- or up-regulated genes.

Gene sets for which Gene Ontology (GO) molecular functions were enriched are shown on the X-axis (including the corresponding cell type name followed by ‘down’ for downregulated and ‘up’ for upregulated genes) with the corresponding GO terms shown on the Y-axis. The sizes of the dots are proportional with the gene ratio which corresponds to the number of genes associated with a given term divided by the number of all genes associated with this term. The colors of dots indicate the level of significance of the adjusted p-value with a gradient from blue (marginally significant) to red (highly significant).

**Figure 4c.**
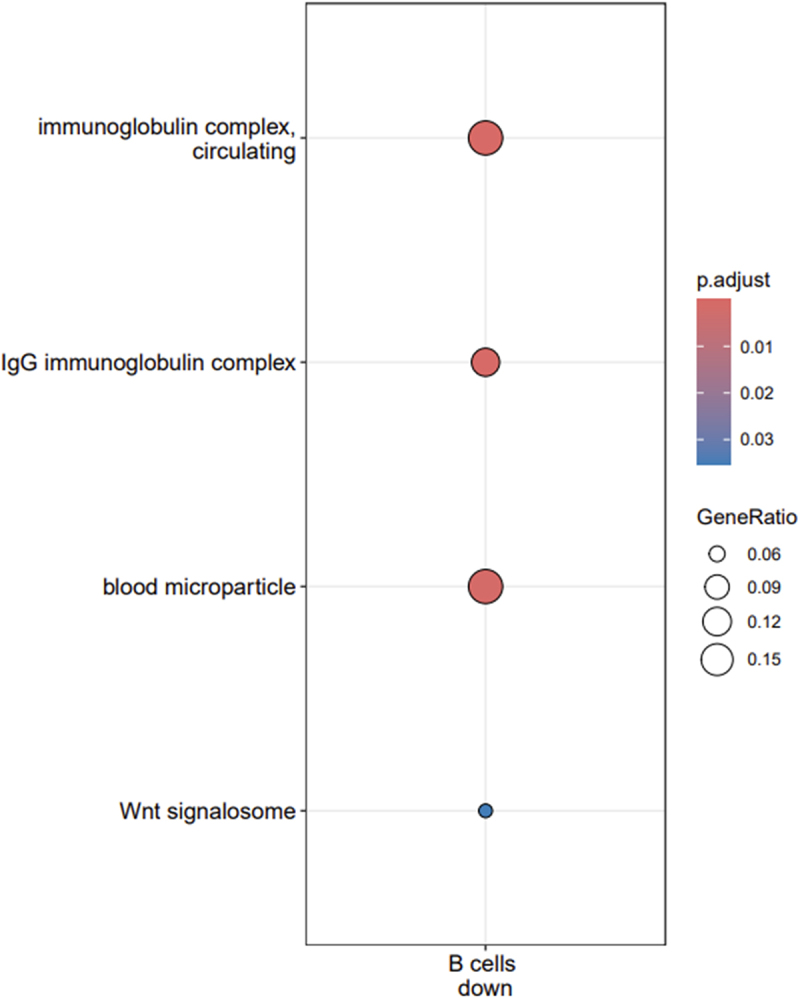
Enrichment of cellular compartment go terms of downregulated genes in B cells.

Enriched GO terms for downregulated genes in B cells are shown on the Y-axis. The sizes of the dots are proportional with the gene ratio which corresponds to the number of genes associated with a given term divided by the number of all genes associated with this term. The colors of dots indicate the level of significance of the adjusted p-value with a gradient from blue (marginally significant) to red (highly significant). Note that there were no enriched terms in the other gene sets.

**Figure 4d.**
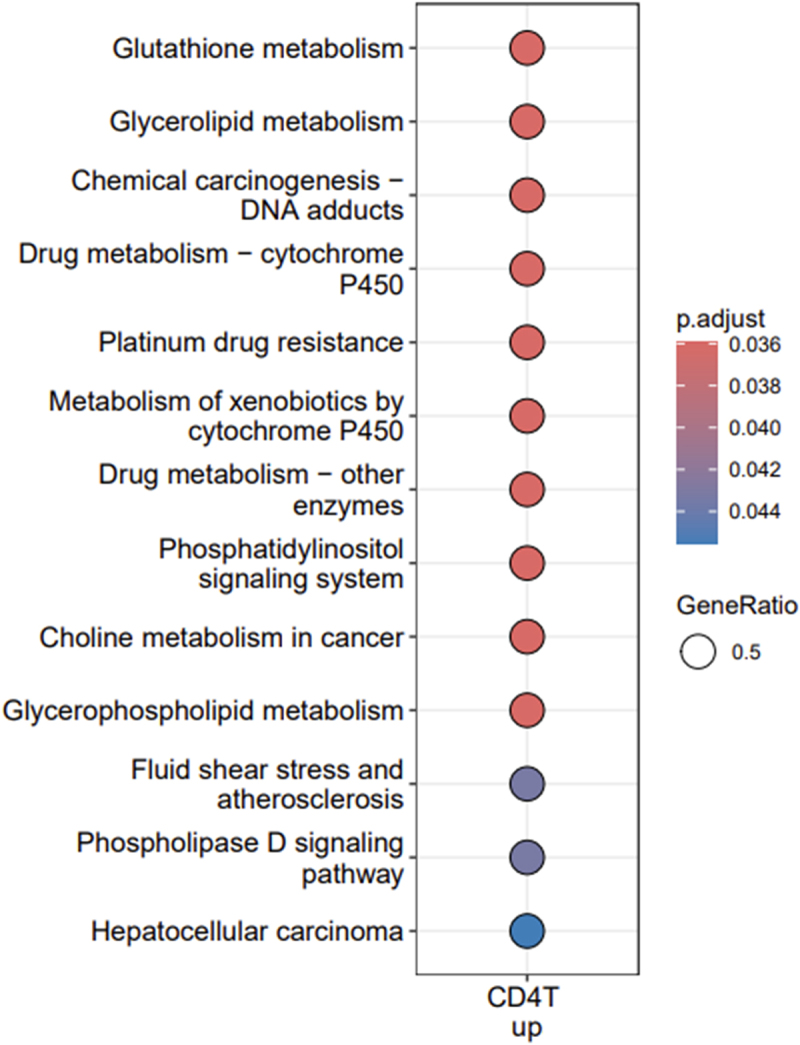
Enrichment of KEGG pathways of upregulated genes in CD4 T cells.

Enriched pathways of the Kyoto Encyclopedia of Genes and Genomes (KEGG) for upregulated genes in CD4 T cells are shown on the Y-axis. The sizes of the dots are proportional with the gene ratio which corresponds to the number of genes associated with a given pathway divided by the number of all genes associated with this pathway. The colors of dots indicate the level of significance of the adjusted p-value with a gradient from blue (marginally significant) to red (highly significant). Note that there were no enriched terms in the other gene sets.

## Discussion

Among adults from a population-based Swiss cohort, 29.4% were classified with high AL using an AL score based on the sum of dichotomized values with cutoffs defined by clinical criteria. A higher proportion of males than females was found in the high AL group, which is consistent with previous findings indicating a higher AL in males [48]. Furthermore, the high AL group was older than the low AL group, consistent with stronger AL effects expected in older people because of ‘wear and tear.’ All biomarker values were significantly higher in the high AL group, except for HDL cholesterol which was significantly lower. This is to be expected as this is the only biomarker for which values below, and not above, the cutoff value contributes to higher AL. Overall, 263 functional CpG-gene pairs were identified in response to high AL as compared to low AL, corresponding to 250 unique CpGs and 138 DMGs across six cell types. More than two-thirds of DMGs were found in CD8 T cells or neutrophils, indicating that DNA methylation effects produced by AL primarily affected these two blood cell types. Cell-type specific differences in response to AL were observed in both the methylation statuses of differentially methylated CpG sites as well as in the ratios of downregulated/upregulated genes for DMGs. Furthermore, among enriched GO BPs terms found for downregulated genes of CD8 T and B cells, many were linked to the adaptative and the humoral immune responses, respectively.

The AL construct considers dysregulation of the neuroendocrine, immune, cardiovascular and metabolic systems, with biomarkers of the neuroendocrine response acting as primary mediators [5]. Although neuroendocrine axis markers, such as adrenaline or noradrenaline, were absent from the AL score used in this study, several DMGs were previously reported to be associated with potential negative neuroendocrine effects, which could theoretically contribute to higher AL. For example, in a genome-wide association study (GWAS) of 53,400 people with irritable bowel syndrome, DOCK9 was associated with mood and anxiety disorders [49]. In addition, BCL11B has been previously shown to be critical for neurodevelopmental transcription with implications in multiple diseases of the central nervous system such as Huntington’s disease and Alzheimer’s disease [50–52]. Variants of LRPAP1 have similarly been linked to susceptibility to degenerative dementia [53]. However, further studies will be required to assess the potential contribution of these genes to the neuroendocrine component of AL. Interestingly, the gene ontology biological term ‘Forebrain generation of neurons’ was enriched among downregulated genes of CD8T cells, which may relate to similar gene expression changes occurring within the brain since both acute and chronic stress can suppress neurogenesis in the hippocampus [54]. It remains to be determined whether the genes identified also play a role in the brain in response to AL. Finally, although previously reported loci in GWASs in whole blood samples for post-traumatic stress disorder [55] or depression [56] - both of which are associated with elevated AL [21,57–60] - were not detected in the present study, this is possibly due to the limited number of individuals (*N* = 2 per group) reporting to have suffered from depression in the SKIPOGH cohort. Regardless, VWDE – a DMG identified in the present study – was previously found to be differentially methylated in Tutsi pregnant women exposed to the genocide in Rwanda relative to controls [61]. Moreover, the differentially methylated CpG site cg06484146 associated to the VWDE gene in the present study was also differentially methylated in the study of Tutsi mothers exposed to the genocide and in their offspring. However, the sample size of this previous study was relatively small, warranting further investigation. Despite the evidence from previous studies, it is important to note that the role or relative expression of the genes cited may have been previously determined in specific tissues and that their function and gene expression pattern in whole blood samples may differ and/or not be relevant to AL.

With regards to the immune system, more than 20 immune-related DMGs were identified. For example, CD27 is involved in T and B cell activation [62], CD96 participates to CD8 T cell activation and effector responses [63], CD8B is part of the CD8 receptor complex which participates in T-cell type differentiation [64], and ITK is an important modulator of T-cell signaling and function [65,66]. In addition, RHOH is involved in T-cell receptor signaling and in regulation of the antibacterial inflammatory response [67,68], while TRBC2 codes for one of the β-chain constant region variants of the T-cell receptor [69]. Finally, NLRP2 is a novel NOD-like receptor which plays a critical role in the progress of immune responses and is a potential regulator of inflammatory signals [70]. Adding to this evidence, enriched GO BP terms of downregulated genes of CD8 T and B cells were linked to the adaptive and humoral immune responses. This is consistent with the fact that high allostatic load is associated with a dysregulated immune system and, since enriched BP terms were found for downregulated genes, that the immune response could be suppressed [71–75]. In contrast to previous reports showing associations between high AL and chronic low-grade inflammation [72,73,75,76], inflammation-related processes were not found among enriched GO terms in this study. This could be due to the SKIPOGH population being relatively healthier, thus contributing to limited variation of CRP. To test whether CRP alone under-detected chronic low-grade inflammation, analyses were repeated using only CRP at a cutoff of 3 mg/L (i.e., the same cutoff value as that used to compute the full AL score) to distinguish low (*N* = 354) from high (*N* = 75) CRP samples. This did not yield inflammation-related terms in gene ontology enrichment results, indicating that CRP levels did not reflect chronic low-grade inflammation in this subset of patients (data not shown).

Several cardiovascular-related DMGs were identified in the present analyses, which could potentially correspond to the cardiovascular component of AL. For instance, SPEG is a key regulator of cardiac calcium homeostasis [77–79], ADAM12 inhibition prevents cardiac hypertrophy and its expression has been found to be increased in hypertrophic obstructive cardiomyopathy [80], ITGA6 was shown to be upregulated in patients with peripheral arterial disease [81], and RHOH is involved in coronary artery disease [82]. Other notable DMGs include SPTBN1, FBLN2, and PHGDH. SPTBN1 was identified as a new potential regulator of the leaky phenotype of atherosclerotic plaque microvessels [83]; and FBLN2 encodes the extracellular matrix protein fibulin 2 which may have an important role in the progression of atherosclerosis in females and may serve as a biomarker for atherosclerosis [84,85]. PHGDH overexpression was previously shown to repress the calcification of human coronary artery vascular smooth muscle cells [86] and variants of the OBSCN gene have been associated with different cardiomyopathies [87–89].

The final system covered by the allostatic load concept, the metabolic system, was also reflected in the results from this study. For example, in a GWAS of clustering of metabolic phenotypes, PLCG1 variants were associated with metabolic syndrome [90]. In addition, SLC2A9 single nucleotide polymorphisms (SNPs) and plasma uric acid were associated with obesity; results that were further replicated [91]. Finally, C1orf145 was associated with blood pressure [92], and SPEG appears to participate in diabetic cardiomyopathy through insulin resistance [79].

In addition to risk of cardiovascular disease and risk of premature mortality, AL has been linked to cancer risk, aggressive tumor characteristics and shorter survival among cancer patients [93–97]. Interestingly, many of the DMGs identified had multiple roles and several were potential cancer biomarkers or were previously shown to be implicated in various types of cancers, including ABLIM1 [98], ADAM12 [99–101], ARHGEF4 [102], FBLN2 [103,104], ITGA6 [105,106], LRPAP1 [107], Ly9 [108], NT5E [109], PITPNC1 [110,111], PLCG1 [112–114], OBSCN [115,116], RHOH [117,118], SPTBN1 [119,120], SPOCK2 [121] and SPRY1 [122]. However, it should be noted that many of these genes play multiple roles outside cancer and that their presence in this study does not necessarily mean that they are indicative of cancer. Hearing loss is another condition which has also been associated with higher allostatic load in previous studies [123]. DMGs for which gene variants were identified in association with hearing loss were also identified, including COL9A3 [124] and SLC7A8 [125]; CACHD1 was also identified in connection to hearing loss in mice [126]. Another interesting DMG was DNMT3a which is a DNA methyltransferase. It has been shown that it can both repress or promote gene expression in neurogenic genes and is expressed in the central nervous system [127,128]. The role of DNMT3a is broader as it can affect the methylation of multiple regions in the genome [129], plays a role in multiple cancer types [130–132] and is essential for hematopoietic stem cell differentiation [133]. It is also worth mentioning that the DMG BACH2 is a marker of DNA damage and aging [134] since both of these processes could contribute to AL.

### Strengths and limitations

Strengths of this study include the simultaneous analysis of transcriptome and DNA methylation analysis and the use of signal deconvolution methods to estimate cell-specific signals. Signal deconvolution enables higher granularity from bulk data. For example, a previous study observed no significant DNA methylation changes in association with methotrexate treatment response in rheumatoid arthritis when using whole blood analyses. By contrast, when applying TCA and performing cell-type specific analyses, differential methylation was found in association with treatment response [135]. Importantly, TCA produces less robust estimates of cell-type-specific signals for cell types with low proportions [30]. This could potentially lead to less reliable results for CD8 T cells than for neutrophils, the latter corresponding to the most abundant cell type in whole blood (Supplementary Figure S1). Although estimates obtained with TCA did not have a control in this study, e.g., data from physically sorted cells with flow cytometry, the fact that the results showed some cell-type specific genes and pathways corresponding to the correct cell types suggests that the deconvoluted signals were indeed attributed to the correct cell types. For instance, the CD8 beta subunit (CD8B) gene was detected in CD8 T cells, CRTAM which determines the CD4+ cytotoxic T lymphocyte lineage [136] was found in CD4 T cells, the transcription factor EBF1 which is essential for the maintenance of B cell identity [137] and B cell scaffold protein with ankyrin repeats (BANK1) were detected in B cells. Similarly, GO enrichments detected among DMGs included T cell differentiation and activation in CD8 T downregulated DMGs, and B cell receptor signaling as well as B cell activation in DMGs downregulated in B cells.

Several limitations must also be acknowledged. First, there is no consensus on which biomarkers should be included in allostatic load (AL) scores or how they should be weighted and combined. This lack of standardization makes it challenging to compare AL measures across studies [138,139]. Because the AL biomarker index used for scores relies on dichotomized values and the distribution of low vs high AL groups is skewed, there may be a reduction of sensitivity. Analyses were hence repeated using an alternate method to compute the AL score: a sum of the Z-standardized values for each biomarker (hereafter ‘AL scores based on Z-scores,’ see Materials and methods for details) which uses cohort specific thresholds instead of clinical values. Overall, 72% of the unique DMGs and 59% of the unique functional CpG sites identified using AL scores based on Z-scores were identical to those found previously using the AL biomarker index based on dichotomized values (Suplementary Table S3). GO enrichment results with AL scores based on Z-scores also showed immune-related terms in common with those previously found in the gene categories with the highest number of genes (i.e., downregulated genes in B cells and in CD8T) but also revealed additional gene categories in other cell types with enrichments (Supplementary Figure S3). Of note, the term ‘Serotonin metabolic process’ was enriched in downregulated genes from monocytes and ‘Cortisol biosynthetic process’ was enriched in CD4 upregulated genes, both of which could affect the neuroendocrine axis of AL. Second, the SKIPOGH population includes only individuals of European descent and relatively healthy individuals; this may limit the generalizability of the results. In particular, the findings of this study may not be recapitulated in cohorts in which participants are more ethnically diverse, exposed to racial inequalities and/or to stronger socioeconomic differences. This limitation may have led to less pronounced differences in AL between the two groups. Consistent with this, chronic low-grade inflammation measured via CRP levels was not detected in high AL individuals in contrast to previous reports [72,73,75,76]. This could be due to only including one biomarker (CRP) for acute inflammation when constructing the AL score, thereby reflecting acute rather than chronic inflammation. Of note, participants with signs of infection had their appointment rescheduled to avoid short-term effects of infections on CRP levels. Results from this study should be validated in other larger, ethnically and socially diverse cohorts to ensure generalizability. From a methodological point of view, the package EpiMix that was used tests for correlations between methylation and transcriptional levels between a test and a control group. Although this permits subset identification of differential methylation in association with gene expression levels, more complex relationships exist between methylation and gene transcriptional levels [140,141] that were not taken into account in this study. Finally, Illumina Infinium MethylationEPIC v1.0 arrays were used, allowing for methylation assessment of about 850’000 CpG sites. However, this is only a small fraction (~3%) of the estimated 28 million CpG sites existing in the human genome. Future accessibility to whole genome bisulfite sequencing or high fidelity long read sequencing technologies may provide further insights [142].

## Conclusion

In the individuals under study, the high AL group consisted of 27% of participants. The integrated analysis of DNAm and transcriptome data has shown that AL has a multi-system impact on gene transcription and DNA methylation across six different cell types. Most genes that were differentially methylated in response to AL were found in CD8 T cells and in neutrophils. Several immune processes were enriched in downregulated genes of CD8 T and B cells, suggesting an immune dysregulation compatible with high AL. Importantly, all four systems composed within the AL construct were represented among the identified DMGs. Although validation in more diverse and larger cohorts is needed, the results of this study may provide biomarker genes for AL as well as potential gene targets to lower AL.

## Supplementary Material

Supplemental Material

## Data Availability

Data for the SKIPOGH cohort is available upon formal request submitted to the SKIPOGH steering committee (https://www.maelstrom-research.org/study/skipogh). Supplementary tables S1 to S3 are available at https://doi.org/10.5281/zenodo.17198739.
