# Supplementary material for: Integrative DNA methylation and transcriptome analysis reveal cell-type specific patterns in response to elevated allostatic load
Source: Epigenetics. 2025 Oct 29;20(1):2578552. doi: 10.1080/15592294.2025.2578552 (PMC12574575; doi:10.1080/15592294.2025.2578552)
Supplement: Supplemental Material [file KEPI_A_2578552_SM0550.docx]

**SUPPLEMENTARY MATERIAL**


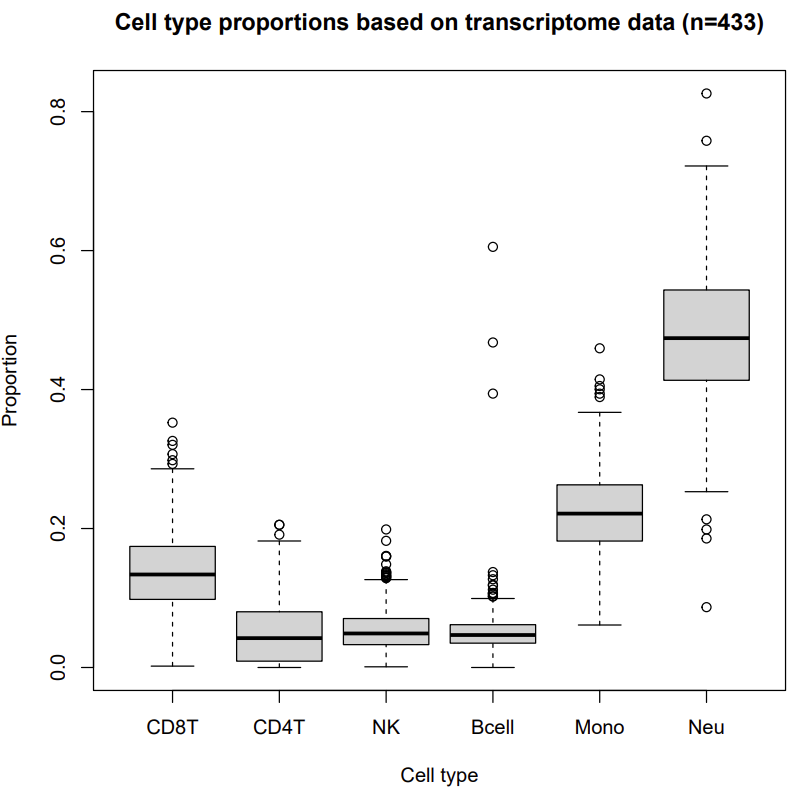


**Supplementary Figure 1.** Cell type proportions based on transcriptome data.

*Boxplots indicating the distribution of the cell type proportions estimated from transcriptome data from all participants (N=429) using CIBERSORTx.*

**
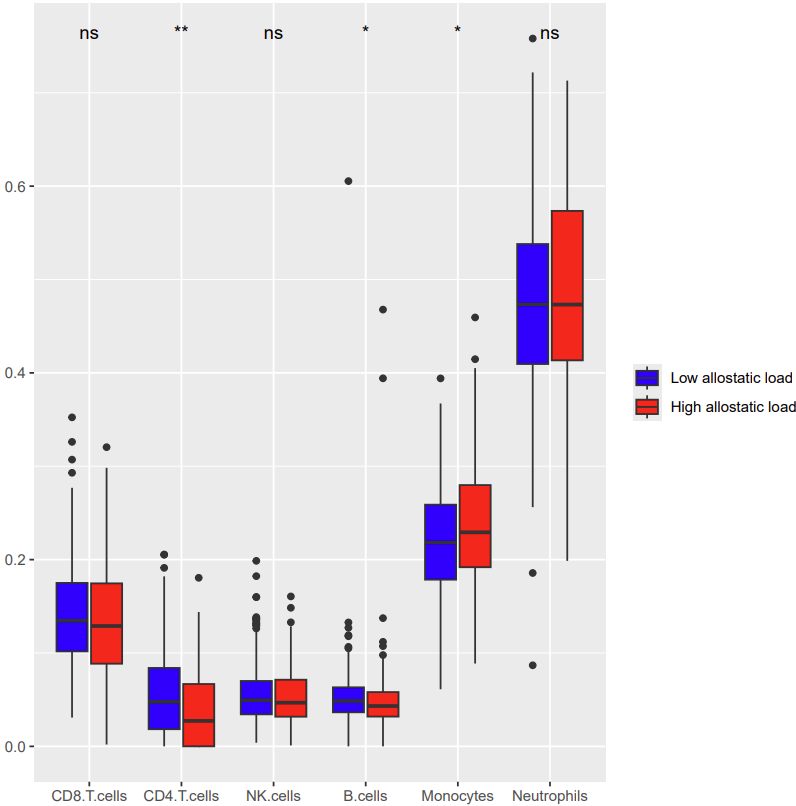
**

**Supplementary Figure 2.** Cell type proportions based on transcriptome data and stratified per allostatic load group (N=303 for low AL group, N=116 for high AL group)

*Boxplots indicating the distribution of the cell type proportions for the low and high allostatic load groups (in blue and red, respectively) estimated from transcriptome data from all participants (N=429) using CIBERSORTx. ns: non-significant, *: p-value < 0.05, **: p-value < 0.01 following Wilcoxon test.*

**
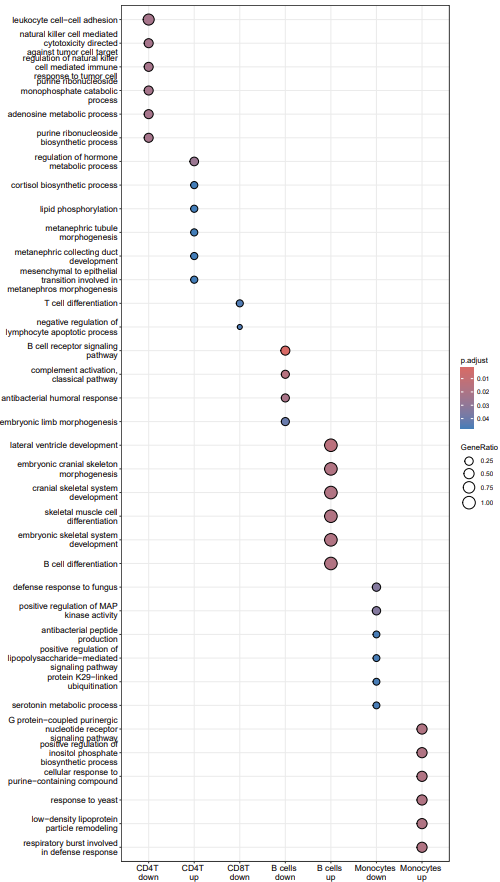
**

**Supplementary Figure 3.**

**Enrichment of biological process GO terms by cell type and down- or up-regulated genes with allostatic load scores based on Z-scores.**

*Gene sets for which Gene Ontology (GO) biological process* *sum-score of absolute z-score values were enriched are shown on the X-axis (including the corresponding cell type name followed by “down” for downregulated and “up” for upregulated genes) with the corresponding GO terms shown on the Y axis. The sizes of the dots are proportional with the gene ratio which corresponds to the number of genes associated with a given term divided by the number of all genes associated with this term. The colors of dots indicate the level of significance of the adjusted p-value with a gradient from blue (marginally significant) to red (highly significant).*

**Table S1. Epimix functional CpG-gene pairs results per cell type and correlation used.**

**Table S2. Gene Ontology (GO) and Kyoto Encyclopedia of Genes and Genomes (KEGG) gene enrichment analyses results per cell type and per gene expression direction.**

**Table S3. Epimix functional CpG-gene pairs results per cell type and correlation used when using AL scores based on Z-scores.**

Supplementary tables S1 to S3 are available at <https://doi.org/10.5281/zenodo.17198739>.
